# Peptide platform for 3D-printed Ti implants with synergistic antibacterial and osteogenic functions to enhance osseointegration

**DOI:** 10.1016/j.mtbio.2024.101430

**Published:** 2024-12-26

**Authors:** Chenying Cui, Yifan Zhao, Jingyu Yan, Ziyang Bai, Guning Wang, Yingyu Liu, Yurong Xu, Lihong Zhou, Kaifang Zhang, Yanling Mi, Binbin Zhang, Xiuping Wu, Bing Li

**Affiliations:** a Shanxi Medical University School and Hospital of Stomatology, Taiyuan, 030001, Shanxi, China; b Shanxi Province Key Laboratory of Oral Diseases Prevention and New Materials, Taiyuan, 030001, Shanxi, China; c Academy of Medical Sciences, Shanxi Medical University, Taiyuan, 030001, Shanxi, China

**Keywords:** 3DTi, Peptide platform, Fusion peptides, Antibacterial, Osseointegration

## Abstract

Bone defects caused by trauma, infection, or tumors present a major clinical challenge. Titanium (Ti) implants are widely used due to their excellent mechanical properties and biocompatibility; however, their high elastic modulus, low surface bioactivity, and susceptibility to infection hinder osseointegration and increase failure rates. There is an increasing demand for implants that can resist bacterial infection while promoting osseointegration. In this study, we developed a peptide platform to engineer a multifunctional 3D-printed Ti implant (3DTi) modified with a fusion peptide composed of minTBP-1 (targeting peptide), KR-12 (antibacterial peptide), and GFOGER (adhesion peptide), termed 3DTi-NFP. This design enables specific targeting, localized delivery, prevention of peptide release into circulation, and functional integrity through linker retention. In both in vitro and in vivo infected bone defect models, 3DTi-NFP implants demonstrated excellent biocompatibility and achieved over 90 % bactericidal efficiency against *S. aureus* and *E*. *coli*. The implants reduced bacterial colonization while enhancing adhesion, proliferation, and differentiation of bone marrow mesenchymal stem cells (BMSCs), significantly upregulating osteogenic genes and protein expression. Transcriptome sequencing further explored the molecular mechanisms underlying the synergistic effects of 3DTi-NFP, revealing activation of the focal adhesion and PI3K-Akt signaling pathways-key contributors to cell adhesion, matrix formation, and new bone formation. Overall, this study provides a promising strategy to improve the long-term success of Ti-based implants, with significant potential for tissue regeneration and clinical applications.

## Highlights

• Created a peptide platform for 3DTi implants with antibacterial and osteogenic effects.
• Used minTBP-1 for specific Ti binding, building a stable, targeted peptide platform.
• Combined KR-12 and GFOGER peptides to enhance osseointegration via synergistic effects.
• Achieved 90 % antibacterial efficacy of 3DTi-NFP against *S. aureus* and *E. coli.*
• Activated focal adhesion and PI3K-Akt pathways to promote osseointegration.

## Graphical abstract

**Figure:**
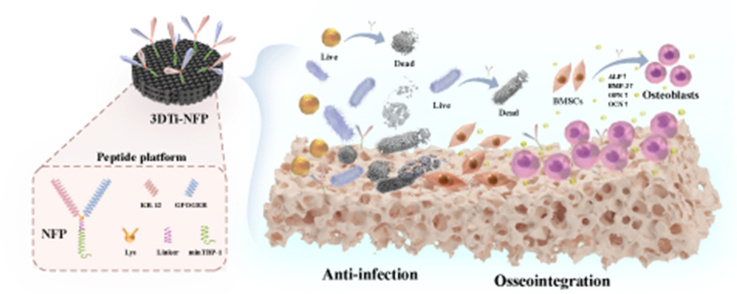

## 1 Introduction

Bone defects caused by trauma, infections, or tumors can significantly impair patients' health and quality of life. Traditional treatment methods, such as medication or bone grafting, often show limited effectiveness and are associated with severe complications [1]. Titanium (Ti) and its alloys have become the standard materials for orthopedic implants due to their outstanding mechanical properties, corrosion resistance, and biocompatibility. However, challenges such as a high elastic modulus, low surface bioactivity, and the risk of postoperative infections often hinder effective osseointegration, increasing the likelihood of implant failure. Research increasingly highlights aseptic loosening and infections as the primary causes of implant failure. To enhance Ti-based implants' long-term stability and success, it is crucial to develop surfaces that promote both osseointegration and antibacterial activity. In this context, surface modification has emerged as a promising strategy to engineer Ti implants by providing dual functions: promoting osteogenesis and preventing bacterial infections [2,3].

Various functional antibacterial strategies have been explored to reduce the risk of bacterial colonization on implant surfaces. Recent approaches include surface modification with sharp morphologies for physical sterilization [[4], [5], [6]], antibiotic loading (e.g., vancomycin [7,8], tinidazole [9]), the application of bioactive molecules (such as antimicrobial peptides (AMPs) [[10], [11], [12], [13]] or nano-coatings of noble metals [2]), and near-infrared adjuvant therapy [[14], [15], [16]]. Among these strategies, AMPs have gained considerable attention as natural antibacterial agents due to their excellent biocompatibility, low potential for drug resistance, and broad-spectrum antibacterial activity. The KR-12 peptide, comprising residues 18–29 (KRIVQRIKDFLR), represents the smallest active motif derived from the only known human cathelicidin, LL-37. Notably, KR-12 retains the broad-spectrum antibacterial efficacy of LL-37 while minimizing the risks of cytotoxicity and hemolysis associated with the full-length peptide, and lowering production costs [17]. The bactericidal mechanism of the KR-12 peptide is closely linked to its *α*-helical conformation and positive charge. Electrostatic interactions target negatively charged bacterial membranes and disrupt their structure using the “carpet model”, thereby exerting its antibacterial effects [[18], [19], [20]]. Beyond its antibacterial activity, KR-12 exhibits additional biological functions, including immune modulation [21,22], promoting epithelial tissue formation [23], and facilitating bone regeneration [24,25]. Importantly, research has shown that KR-12 can promote osteogenic differentiation in human bone marrow stem cells (hBMSCs) by activating the BMP/SMAD signaling pathway, highlighting its promising application in bone tissue regeneration.

While preventing bacterial colonization, AMPs may also interfere with the adhesion of host cells, thereby hindering the rapid integration of implants with host tissue. A key challenge in the biomedical field is how to effectively prevent bacterial infections without compromising the physiological function of osteoblasts. Therefore, achieving a balance between bacterial inhibition and host-cell compatibility is crucial for successful osseointegration. The ideal implant surface should suppress bacterial adhesion while simultaneously promoting the adhesion and proliferation of host cells [26].

Facilitating the interaction between host cells and the extracellular matrix (ECM) can direct specific cellular behaviors [[27], [28], [29]]. The *α*2*β*1 integrin, a protein receptor abundantly expressed on the surface of osteoblasts, regulates cell adhesion, migration, proliferation, and signal transduction by binding to ECM collagen [30]. In particular, the binding of *α*2*β*1 integrin to type I collagen activates signaling pathways that enhance osteoblast differentiation and promote matrix mineralization [31]. GFOGER is a short peptide fragment derived from the *α*1 chain of type I collagen. Its triple helix structure exhibits a high affinity and specificity for the *α*2*β*1 integrin on the cell membrane [32]. In comparison to the commonly used adhesive peptide RGD, GFOGER offers a distinct advantage in osteogenic performance due to its lack of competitive binding sites and its inherent properties that selectively target osteogenic cells [33]. BMSCs, as pluripotent cells, can differentiate into osteoblasts and secrete growth factors that facilitate bone regeneration. This makes them an ideal model for studying the interactions between materials and cells. We anticipate that the application of GFOGER will significantly enhance the osteogenic differentiation of BMSCs. Nevertheless, achieving precise control over the proportions and delivery of multiple peptides on Ti surfaces remains a significant challenge.

To date, numerous research strategies have sought to graft bioactive peptides onto Ti surfaces [34]. These strategies encompass both physical and chemical approaches. However, physical binding often lacks stability or may result in excessive release due to the weak nature of non-covalent bonds. In contrast, the chemical binding process is complex and frequently involves the use of toxic substances [35]. Consequently, the development of a straightforward, stable, and safe multifunctional high-density graft modification method represents an attractive research goal in the field of surface bioengineering.

Recent advancements in phage display and cell surface display techniques have enabled researchers to isolate a peptide known as Mintitanium Binding Peptide-1 (minTBP-1, RKLPDA) [35], which can specifically recognize Ti surfaces without the need for surface pretreatment or harsh reaction conditions. The peptide minTBP-1 binds specifically to the Ti oxide layer through electrostatic interactions. In this binding process, the -O^-^ and -OH_2_^+^ groups in the oxide layer interact with the R1 and D5 residues of minTBP-1, respectively [36,37]. Our objective is to construct a fusion peptide (FP) by conjugating minTBP-1 with the peptides KR-12 and GFOGER, thereby enhancing the osseointegration of Ti implants. In addition, introducing a peptide platform can demonstrate two complementary or synergistic bioactive sequences through precise chemical control [26]. Constructing a peptide platform with orthogonally protected lysine (Lys) as a branching structure, capable of linking two functional sequences (e.g., RGD and PHSRN), has been shown to significantly promote the adhesion, spreading, proliferation, and differentiation of osteoblasts [38,39]. These studies provide a solid foundation for our subsequent experimental design and theoretical analysis. Therefore, could the combination of peptide platform technology with specifically targeted peptides achieve a one-step multifunctional modification of Ti implants and enhance their osseointegration ability?

Three-dimensional (3D) printing technology offers a high degree of freedom and precise customizability, allowing for the design of structures that closely mimic the characteristics of bone. This capability provides an optimal environment for cellular interaction and adaptation. Ti implants produced through 3D printing (3DTi), featuring nanoscale rough surfaces, enhance cell adhesion and effectively reduce the elastic modulus of the implant to levels comparable to those of human bone. This adjustment minimizes the stress shielding effect surrounding the implant [40], thereby decreasing the loss of supporting bone and the rate of implantation failures [41]. Consequently, the integration of 3D printing technology represents a significant advancement in enhancing osseointegration.

**Scheme 1:**
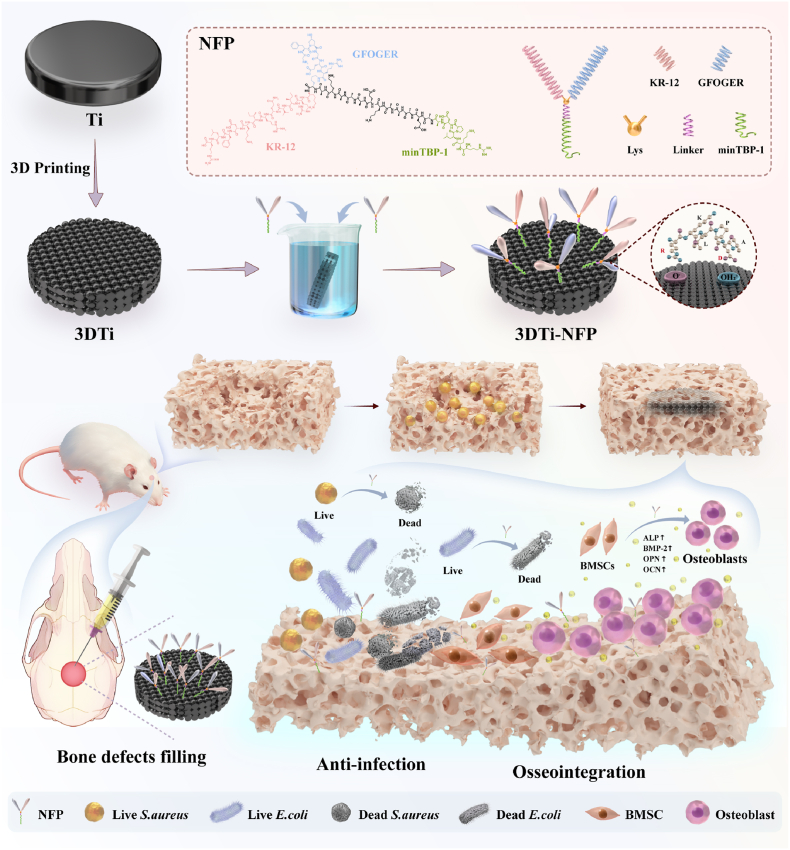
Schematic representation of the design and fabrication of the 3DTi implants functionalized with novel multifunctional FPs for repairing bacterially infected cranial bone defects in a rat model.

In this study, we successfully co-loaded the antibacterial peptide KR-12 and the adhesive peptide GFOGER onto the surface of 3DTi using the specific targeting peptide minTBP-1 and peptide platform technology. This approach allows for localized and targeted effects while preventing the peptides from entering the bloodstream. Additionally, incorporating a linker effectively separates each functional domain, preserving the FP's structural stability and enhancing the functional peptides' efficacy [42]. Importantly, the synergistic interaction between KR-12 and GFOGER may reveal a previously unreported mechanism for enhancing osseointegration. This study systematically investigated the surface morphology, elemental distribution, roughness, wettability, and load efficiency of 3DTi loaded with novel multifunctional fusion peptides (NFPs), as well as their release capacity. Furthermore, the biocompatibility, cell adhesion, antibacterial activity, and osteogenic potential of 3DTi-NFP implants were evaluated using both in vitro and in vivo infected bone defect models. Transcriptome sequencing (RNA-seq) was subsequently performed to analyze relevant cytokines and signaling pathways, aiming to elucidate the mechanism underlying the synergistic promotion of osseointegration. A schematic diagram outlining the complete experimental workflow is provided in Scheme 1. The newly developed multifunctional FP-functionalized 3DTi implant offers localized delivery and targeted release of active peptide molecules, simultaneously exhibiting antibacterial and osteogenic properties to enhance osseointegration around Ti implants.

## 2 Materials and methods

### 2.1 Synthesis of FPs

FP1, FP2, and NFP were synthesized by Qiangyao Biotechnology Co., Ltd. (Shanghai, China) via solid-phase synthesis with Fmoc (9-fluorenylmethyloxycarbonyl) chemistry, following the sequences listed in Table S1. The purity of the non-fluorescent peptides exceeded 99 %, while the purity of the fluorescently labeled peptides (fluorescein isothiocyanate, FITC, green) was above 95 %. The molecular structures of the FPs were predicted and analyzed using the protein analysis software PSIPRED and PEP-FOLD.

### 2.2 Preparation of Ti materials

The Ti substrates (purity 99.89 %) were provided by Jinwu New Material Co., Ltd. (Jiangsu, China) and were prepared in two sizes: 14 mm × 14 mm × 1 mm discs (labeled as Ti/3DTi) for in vitro studies, and 7 mm × 7 mm × 0.5 mm discs for in vivo studies. The Ti substrates were sequentially polished using silicon carbide sandpapers with grit sizes of 400, 800, 1000, 1200, 1500, and 2000. Subsequently, the substrates were ultrasonically cleaned with acetone, 75 % ethanol, and deionized water for 20 min, followed by autoclaving, drying, and disinfection under ultraviolet light for 3 h before use.

### 2.3 Loading and release of FPs

FITC-labeled peptide powders were dissolved in phosphate-buffered saline (PBS) to prepare peptide solutions with 1, 10, 100, and 1000 μg/mL concentrations. These solutions were applied to the 3DTi substrates and incubated overnight at room temperature. The following day, the unbound peptides were washed away with PBS, and the pixel density was quantified by confocal laser scanning microscopy (CLSM, FV1000, Olympus, Japan) to determine the optimal concentration.

To evaluate the specific binding affinity of the FPs to the Ti substrate, FITC-labeled FPs were incubated with Ti, 3DTi, and non-Ti samples in 24-well plates overnight at room temperature. The samples were then examined using CLSM.

The selected optimal concentration was then used to incubate 3DTi substrates to evaluate binding efficiency. A standard curve for FITC-labeled peptides was generated using ultraviolet–visible spectroscopy (UV–Vis). The fluorescence distribution on the 3DTi surface at different time points was monitored using a CLSM, while the fluorescence intensity of the supernatant was measured with UV–Vis. The amount of peptide adsorbed onto different surfaces was quantified based on the standard curve. The excitation and emission wavelengths for FITC were set to 488 nm and 520 nm, respectively. The three modified 3DTi samples were designated as 3DTi-FP1, 3DTi-FP2, and 3DTi-NFP.

To investigate the peptide release behavior, 3DTi-FP1, 3DTi-FP2, and 3DTi-NFP implants were immersed in 1 mL of PBS. The samples were then placed on a shaker set to 300 rpm to ensure constant solution agitation. At designated time points (days 1, 3, 5, 7, 9, 11, 13, 15, and 17), 1 mL of supernatant was collected, and each sample was tested in triplicate. The release profiles of the FPs were analyzed by measuring the fluorescence intensity of FITC in the collected solutions. The amount of peptide released from each sample was quantified by comparing the fluorescence values to a pre-established standard curve, and the release kinetics were modeled using Origin software.

### 2.4 Preparation and characterization of samples

The 3DTi substrates were immersed in 100 μg/mL FP peptide solution at room temperature and incubated for 2 h. After incubation, the treated samples were thoroughly rinsed with PBS before drying in an argon atmosphere. Surface morphology was examined using a field emission scanning electron microscope (FE-SEM, JSM-7001F, JEOL, Japan) at an accelerating voltage of 10 kV. Energy-dispersive X-ray spectroscopy (EDS, QX200, Bruker Optics, Germany) analyzed elemental distribution on the sample surfaces. Surface roughness (Ra) was measured over a 10 μm × 10 μm area using atomic force microscopy (AFM, SPA-300HV, NSK, Japan). Surface wettability was evaluated by the sessile drop method with a contact angle analyzer (JC2000DF, Powereach, China). Additionally, X-ray photoelectron spectroscopy (XPS, K-α, Thermo Fisher, USA) was employed to assess the elemental composition and chemical states on the sample surfaces.

For biomechanical testing, compressive strength assessments of Ti and 3DTi samples were performed on a universal testing machine (Model 880, MTS, USA) according to ISO 13314 standards. The equipment had a maximum load capacity of 500 kN, and the testing speed was set at 0.5 mm/min. Five independent samples were tested for each experiment, with three discs analyzed for each surface treatment.

### 2.5 Cell culture

BMSCs were harvested from the femoral bone marrow of Sprague-Dawley (SD) rats. Cells at the third passage were cultured in Dulbecco's Modified Eagle Medium (DMEM) containing 10 % fetal bovine serum (FBS, Gibco, USA) and 1 % penicillin-streptomycin (Gibco, USA) under standard incubation conditions (37 °C, 5 % CO_2_). For experimental use, BMSCs from passages 4–7 were seeded in 24-well plates at a density of 1 × 10^4^ cells per sample. All in vitro assays were performed in triplicate across three independent replicates to ensure statistical reliability.

#### 2.5.1 Cytocompatibility evaluation of samples

BMSCs proliferation was assessed using the CCK-8 assay (Solarbio, China). On days 1, 4, and 7, 500 μL of a 10 % CCK-8 solution was added to each well, followed by a 2-h incubation at 37 °C. After incubation, 100 μL of supernatant from each well was transferred to a 96-well plate, and optical density (OD) was recorded at 450 nm using a spectrophotometer.

The Live/Dead Cell Staining Kit (CA1630, Solarbio, China) was employed to assess BMSCs viability. After 1 and 3 days of culture, cells were stained following the manufacturer's protocol. In live cells, esterases cleave calcein-AM into calcein, producing green fluorescence, while ethidium homodimer-1 penetrates only the membranes of dead cells, binding to nucleic acids to produce red fluorescence. Finally, fluorescence microscopy was used to observe and capture cell images.

#### 2.5.2 Adhesion activity of BMSCs

Cells were cultured in a serum-free medium for 4 h to investigate BMSCs' adhesion and morphology. Cultured samples were fixed with 4 % paraformaldehyde at room temperature for 30 min. Cells were then permeabilized with 0.1 % Triton X-100 (Beyotime, China) for 5 min. Actin filaments were stained with rhodamine-phalloidin for 30 min in the dark. Finally, cell nuclei were stained with 4',6-diamidino-2-phenylindole (DAPI, 5 mg/mL; Invitrogen, USA) for 10 min. The samples were then examined using CLSM.

### 2.6 Bacterial culture

To assess antibacterial efficacy, *Staphylococcus aureus* (*S. aureus*, ATCC 29213) and *Escherichia coli* (*E. coli*, ATCC 43300) were selected for testing, with initial cultivation performed in tryptic soy broth (TSB). Single colonies from each bacterial species were transferred onto TSB agar plates and incubated overnight. Following incubation, one colony was transferred to TSB and shaken at 220 rpm at 37 °C for 6 h. This bacterial culture was then diluted with sterile PBS to achieve a concentration of 1 × 10^8^ CFU/mL.

#### 2.6.1 Plate count method

The antibacterial activity was assessed by applying a plate-count technique. Initially, a 1 μL bacterial suspension was placed on different substrates and incubated at 37 °C for 12 h. After incubation, bacteria from each group were dislodged by sonication and collected in 1 mL of sterile PBS with 0.1 % Tween-80. The suspension underwent a series of 10-fold dilutions, and 100 μL of each diluted sample was spread onto agar plates. These plates were then incubated at 37 °C for an additional 12 h. CFUs were subsequently counted and documented by photography. The antibacterial efficacy (R) was determined using the formula: R = (A - B)/A × 100 %, where R denotes the antibacterial efficacy, A is the mean CFU count in the control group (Ti), and B is the mean CFU count in the experimental groups (3DTi, 3DTi-FP1, 3DTi-FP2, and 3DTi-NFP).

#### 2.6.2 Inhibition zone assays

Additionally, a 100 mm agar plate was uniformly seeded with a 1 × 10^8^ CFU/mL bacterial suspension using a sterile cotton swab. Each Ti substrate was placed at the center of the plate, with untreated Ti serving as the control. Inhibition zones were observed and photographed after a 12-h incubation at 37 °C.

#### 2.6.3 Live/dead bacterial staining assay

The viability of bacteria was evaluated using a Live/Dead BacLight Bacterial Viability Kit (Thermo Fisher, Shanghai, China). After incubation at 37 °C for 12 h, bacterial suspensions were rinsed with PBS. Next, a staining solution comprising 10 μM propidium iodide and 10 μg/mL SYTO9 was applied, followed by an additional 30-min incubation. Finally, bacterial viability on the substrate surfaces was observed and documented via CLSM.

#### 2.6.4 Bacterial adhesion and morphology observation

Bacterial morphology on distinct Ti surfaces was examined with FE-SEM. A 1 mL bacterial suspension was added to various substrates arranged in a 24-well plate, followed by incubation at 37 °C for 12 h. The samples were then fixed in 2.5 % glutaraldehyde at 4 °C for 4 h. Next, the samples were dehydrated in a graded ethanol series ranging from 30 % to 100 % and subsequently air-dried. Following gold sputter coating, the bacterial morphology was visualized under FE-SEM.

### 2.7 Osteogenesis in vitro

#### 2.7.1 Alkaline phosphatase (ALP) activity measurement

ALP activity in BMSCs was analyzed using a 5-bromo-4-chloro-3-indolyl phosphate/nitro blue tetrazolium (BCIP/NBT) staining kit and an ALP assay kit (Beyotime, China). BMSCs were cultured on Ti surfaces in an osteogenic medium. After 7 and 14 days of incubation, BMSCs were fixed in 4 % paraformaldehyde for 30 min. BCIP/NBT staining solution was then added, and samples were incubated for 1 h to enable ALP visualization. Following staining, the BCIP/NBT solution was discarded, and samples were rinsed with PBS to stop the reaction. ALP-stained images were obtained using an optical microscope.

For quantitative analysis of ALP activity, BMSCs were lysed on ice with 1 % Triton X-100 for 30 min. The lysate was centrifuged at 12,000 rpm for 4 min at 30 °C, and the resulting supernatant was collected. A 50 μL portion of this supernatant was mixed with 50 μL of ALP assay working solution and further incubated for 30 min. ALP activity was recorded at an absorbance of 405 nm using a microplate reader.

#### 2.7.2 Alizarin red S (ARS) staining

At 14 and 21 days of culture, cells were fixed in 4 % paraformaldehyde for 15 min, then stained with 0.1 % ARS (Solarbio, China) at 37 °C for 30 min. Following staining, cells were rinsed with PBS and visualized using an optical microscope. For quantification, the mineralized deposits were dissolved in 10 % cetylpyridinium chloride (Sigma-Aldrich, South Korea), and absorbance was recorded at 570 nm with a microplate reader.

#### 2.7.3 Immunofluorescence staining

BMSCs cultured on various sample surfaces for 14 days were fixed in 4 % paraformaldehyde, permeabilized with 0.1 % Triton X-100, and blocked with 5 mg/mL bovine serum albumin (BSA, Beyotime, China) to minimize nonspecific binding. Samples were subsequently incubated overnight at 4 °C with primary antibodies targeting vinculin (VIN, Abcam, UK), ALP (Abcam, UK), bone morphogenetic protein-2 (BMP-2, Abcam, UK), osteopontin (OPN, Affinity Biosciences, USA), and osteocalcin (OCN, Affinity Biosciences, USA). After primary antibody incubation, samples were treated with a fluorescent secondary antibody (Alexa Fluor 488, Abcam, UK) in the dark for 1 h at room temperature. Actin filaments were stained with rhodamine-phalloidin, cell nuclei were labeled with DAPI, and fluorescence images were captured using CLSM.

#### 2.7.4 Real-time quantitative reverse transcription-polymerase chain reaction (qRT-PCR)

To evaluate osteogenic differentiation in BMSCs, the gene expression levels of ALP, BMP-2, OPN, and OCN were analyzed via qRT-PCR. BMSCs were cultured for 14 days in various conditions. Total RNA was isolated with TRIzol reagent (Beyotime, China), and complementary DNA (cDNA) was synthesized using the RevertAid First Strand cDNA Synthesis Kit (Thermo Scientific, USA). qRT-PCR was performed on an ABI Prism 7300 Thermal Cycler (Applied Biosystems, Australia) with SYBR Green as the detection dye. Gene expression was normalized to glyceraldehyde-3-phosphate dehydrogenase (GAPDH) as the reference gene. Relative expression levels were determined using the 2 ^−ΔΔCt^ method, where ΔCt is the average Ct value adjusted to the reference gene. Primer sequences are provided in Table S3.

### 2.8 RNA-seq and data analysis

RNA-seq and bioinformatic analyses were conducted to investigate potential mechanisms underlying the observed effects. BMSCs were cultured on Ti and 3DTi-NFP surfaces and, after 14 days of osteogenic induction, total RNA was extracted using TRIzol reagent. RNA sequencing libraries were constructed and sequenced on the BGISEQ-500 platform from BGI Genomics (Shenzhen, China). Genes showing a fold change above 1.5 or below −1.5 with a *p*-value less than 0.05 were identified as differentially expressed genes (DEGs). Functional annotations of genes and gene products were obtained using Gene Ontology (GO) (http://www.geneontology.org). Bioinformatic analyses were performed via the Bio-Cloud platform (https://bio-cloud.aptbiotech.com/tool).

### 2.9 In vivo study

#### 2.9.1 Implantation operation

The Animal Ethics Committee of Shanxi Medical University Medical Center approved animal experiments in this study. All procedures were conducted following the standards described in the guidelines for the care and use of laboratory animals. Ethics number: KQDW-2024-004. A total of 20 healthy male SD rats weighing approximately 300 g were used to construct 8 mm cranial defect models, with 5 rats in each group.

All rats were anesthetized by intraperitoneal injection of 2 % pentobarbital sodium (2 ml/kg body weight). An 8 mm drill was used to create a defect at the cranial suture, removing the full thickness of the bone. The 3DTi substrates loaded with the FPs were then randomly implanted into the defect site, with unmodified 3DTi implants used as the control group. Following injection of the *S. aureus* suspension, the muscle and skin were sequentially closed using absorbable 4-0 surgical sutures. After 8 weeks, all rats were euthanized by overdose anesthesia, and the calvarial samples were harvested, cleared of all soft tissue, and fixed in 4 % paraformaldehyde.

#### 2.9.2 In vivo antibacterial analysis

Three days post-surgery, secretions from the implantation sites were collected from three randomly selected SD rats in each group using sterile swabs to assess peri-implant infection. Bacterial suspensions from different groups were transferred into 1 mL of sterile PBS with 0.1 % Tween 80 and subjected to ultrasonic treatment. Tenfold serial dilutions (100 μL) were spread on agar plates, and CFUs were counted and documented.

#### 2.9.3 Evaluation of biocompatibility in animals

At 8 weeks post-implantation, blood samples were collected from the heart for routine hematological analysis. Additionally, the heart, liver, spleen, lungs, and kidneys were harvested and subjected to hematoxylin and eosin (H&E) staining to evaluate systemic infection status.

#### 2.9.4 Micro-computed tomography (Micro-CT) detection

To assess bone formation, twenty circular regions of interest, each 50 μm in diameter, were selected around each implant, and micro-CT scans were conducted at high resolution using the Bruker Skyscan 1172 system (Kontich, Belgium). Following scanning, data were reconstructed in 3D, and quantitative analysis of the micro-CT images was performed to determine fluorescence density, bone mineral density (BMD), bone volume-to-total volume ratio (BV/TV), trabecular number (Tb.N), and trabecular thickness (Tb.Th).

#### 2.9.5 Histological analysis

After being fixed in 4 % paraformaldehyde for 48 h, specimens were embedded in resin through a light-curing process. Tissue blocks were then cut into 200 μm sections using the EXAKT300CP hard tissue microtome (EXAKT, Germany) and subsequently ground down to a thickness of 20 μm with the EXAKT400S grinding system (EXAKT, Germany). The sections were stained using H&E and Masson-Goldner's trichrome, and images were captured with a microscope.

### 2.10 Statistical analysis

All experiments were performed in triplicate unless stated otherwise. Data are presented as the mean ± standard deviation (SD). Statistical analyses and quantitative imaging were conducted using GraphPad Prism 9.5 (GraphPad, USA). Statistical significance was assessed using the *t*-test and one-way ANOVA, followed by Tukey's post hoc test, with *p*-values <0.05 (∗) considered significant.

## 3 Results and discussion

### 3.1 Design and synthesis of FPs

**Fig. 1:**
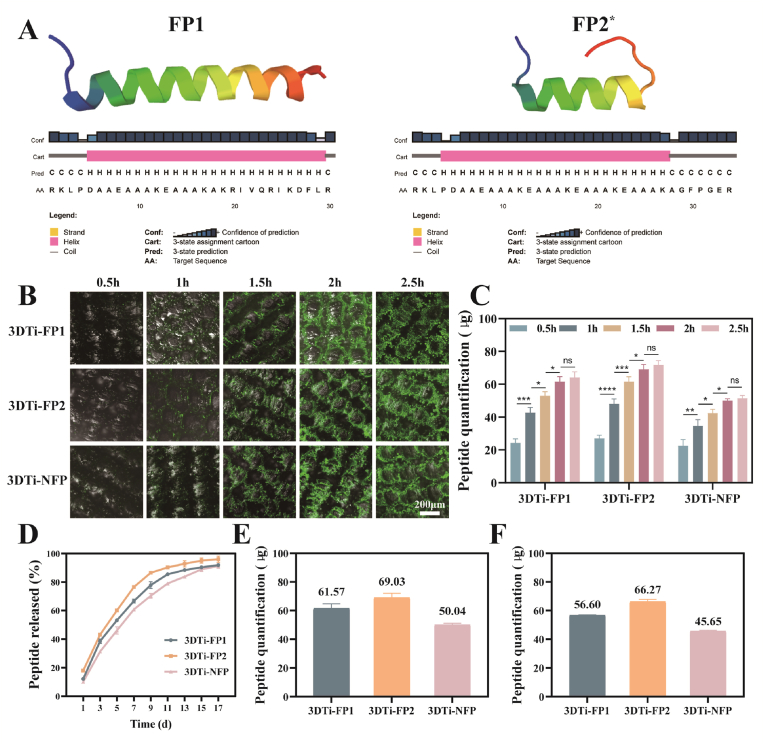
Structure, loading, and release of the FPs. (A) Prediction of the secondary and tertiary structures of FP1 and FP2 peptides using PSIPRED and PEP-FOLD software. (B) Visualization of FITC-labeled peptides loaded onto the 3DTi surface. (C) Quantification of peptide loading on the 3DTi surface at different time points. (D) Cumulative release profile of FPs over 17 days in PBS. (E) Peptide content on 3DTi surface measured after 2h load saturation. (F) Peptide content released on 3DTi surface at 17 days. Data were presented as mean ± SD. ∗*p* < 0.05, ∗∗*p* < 0.01, ∗∗∗*p* < 0.001, ∗∗∗∗*p* < 0.0001, and ns: no significance. (∗The linker of FP2 is extended to (EAAAK)_4_ due to the need for structural prediction. P=Proline, O=Hydroxyproline).

The sequences of the proposed FP platform (Table S1), along with their key structural features, are illustrated in Scheme 1. This platform is composed of four primary components: (i) the bioactive peptide sequences KR-12 and GFOGER; (ii) the linker unit; (iii) lysine residues; and (iv) the specific anchoring group minTBP-1. This study represents the first integration of the antibacterial peptide KR-12 with the adhesion peptide GFOGER, aimed at investigating the effects of these two active motifs on cellular behavior after grafting onto 3DTi materials. To maintain an optimal spatial distance between the functional peptides and the 3DTi substrate while ensuring sufficient proximity for the peptides to exert their stable bioactivity, a rigid spacer unit composed of a high number of hydrogen bonds, EAAAK, was selected [43]. The introduction of lysine residues is crucial for synthesizing a branched conformation, enhancing both the accessibility and orientation of the two functional peptides compared to a linear structure [38]. The inorganic anchoring peptide minTBP-1 exhibits a specific affinity for Ti, enabling rapid and precise recognition and anchoring to the Ti substrate, thereby facilitating the effective loading of the FPs onto the Ti surface in a one-step process. As depicted in Fig. 1A, the secondary structures of FP1 and FP2 consist of random coils and *α*-helices. The random coil structure allows the FPs to exhibit a broader range of motion to interact at the 3DTi interface, while the shared helical structure enhances their functional activity.

### 3.2 Binding and release ability of FPs on 3DTi surface

Using the solid phase synthesis method and referring to the sequence structure in Table S1, three FPs were synthesized successfully in this study. While optimizing peptide binding concentrations, the fluorescence signal of FITC-labeled peptides on the 3DTi surface increased with higher peptide concentrations (Fig. S1). No significant difference in fluorescence intensity was observed between 100 μg/mL and 1000 μg/mL, leading to the selection of 100 μg/mL as the initial incubation concentration for subsequent experiments. The three peptides were rarely observed on non-Ti substrates but were widely distributed across Ti and 3DTi substrates (Fig. S2).

FITC-labeled FPs were then used to visualize and quantify peptide loading efficiency. As shown in Fig. 1B, the fluorescence distribution on 3DTi surfaces loaded with different FPs was uniform. Quantitative analysis of peptide content for various experimental groups was conducted, with the standard curve for peptide quantification illustrated in Fig. S3. The peptide content on the surface increased over time (Fig. 1C), reaching a plateau at 2 h, indicating saturation and the high loading efficiency of the FPs. The amounts of FP1, FP2, and NFP adsorbed onto the 3DTi surface were quantified as 61.57 μg, 69.03 μg, and 50.04 μg, respectively (Fig. 1E). Furthermore, the loading of the newly designed multifunctional FPs was not adversely affected at any of the tested time points.

The cumulative release profile of the FPs over 17 days exhibited a distinctive biphasic release pattern, comprising an initial rapid release phase and a subsequent sustained release phase (Fig. 1D). During the early stage, the rapid desorption of loosely bound peptides on the surface led to a sharp increase in peptide concentration, providing potent antibacterial activity and facilitating osteoblast adhesion in the early treatment phase. In the subsequent phase, the release became diffusion-controlled, driven by the gradual liberation of peptides strongly adsorbed onto the 3DTi surface. This sustained release effectively prevented reinfection and maintained osteogenic activity over an extended period. By day 17, the cumulative release amounts of FP1, FP2, and NFP peptides reached 56.60 μg, 66.27 μg, and 45.65 μg, respectively, achieving near-complete release (Fig. 1F). In addition, this indirectly proves that the binding of FPs to Ti surfaces remains stable even under dynamic conditions, ensuring extended function and efficacy in potential biomedical applications.

The study revealed that this release behavior was predominantly governed by the concentration gradient. As the peptides diffused into the surrounding medium, the concentration gradient diminished, resulting in a gradual deceleration of the release rate. As demonstrated in previous studies [44], the superior fit of the first-order kinetic model further validates the fit of the release system to the physiological conditions (Fig. S4). These findings suggest that the sustained release of FPs provides prolonged bioactive support for bone regeneration, aligning well with the therapeutic objective of enhancing osseointegration for Ti-based implants.

### 3.3 Characterization of FPs binding to 3DTi surface

**Fig. 2:**
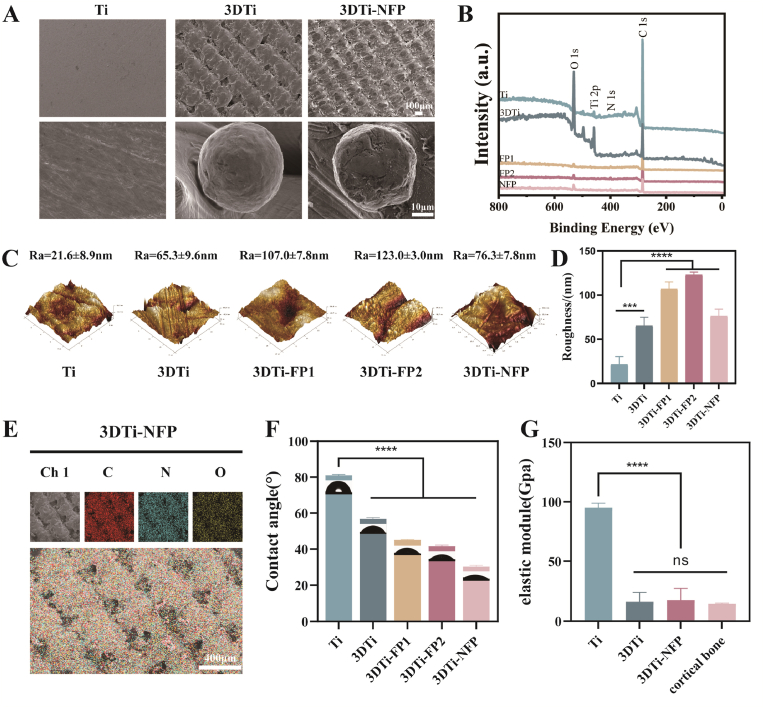
Characterization of modified Ti substrates. (A) SEM images of Ti substrates treated with different modification methods. (B) XPS spectra of Ti substrates after different modifications. (C) AFM images showing the surface morphology of different samples. (D) Surface roughness measurements of the Ti substrates. (E) EDS spectrum capturing the surface of 3DTi-NFP samples. (F) Water contact angle images and quantitative results for each modification. (G) Elastic modulus of Ti substrates following different modifications. Data were presented as mean ± SD. ∗*p* < 0.05, ∗∗*p* < 0.01, ∗∗∗*p* < 0.001, ∗∗∗∗*p* < 0.0001, and ns: no significance.

Subsequently, SEM was used to examine the surface morphology of each sample (Fig. 2A). The smooth Ti surface displayed a relatively flat structure with minor scratches, whereas the 3DTi surface exhibited a network of micropores, with Ti powder particles visible at higher magnification. After incubation of 3DTi with the NFP peptide solution, SEM images showed a uniform distribution of NFP across the 3DTi surface, forming small granular structures without altering the original surface morphology.

XPS analysis revealed the elemental characteristic peaks on each sample surface (Fig. 2B), including Ti 2p (456.0 eV), O 1s (530.8 eV), C 1s (284.8 eV), and N 1s (399.9 eV). After the FPs were bound to the 3DTi surface, the signal intensities of Ti and O decreased, while those of C and N increased. The N/Ti atomic ratio rose from 0.217 in bare Ti to approximately 15.304 in 3DTi-NFP (Table S2) confirming the successful attachment of the peptide coating. The C content observed in the Ti and 3DTi control samples was attributed to organic contaminants from the atmosphere, along with traces of N contamination. Notably, the N peak at 399.9 eV was ascribed to amide groups and other N-containing functional groups characteristic of the peptide sequence. This effect was particularly pronounced in the NFP platform, consistent with its larger molecular weight. Additionally, EDS of 3DTi-NFP samples showed a uniform distribution of Ti, O, C, and N on the surface (Fig. 2E).

AFM was used to assess the morphology and roughness of different Ti surfaces (Fig. 2C and D). The results indicated that the roughness of the Ti surface was 21.6 ± 8.9 nm, while the roughness of the 3DTi surface was significantly higher at 65.3 ± 9.6 nm. After loading with FPs, the roughness of 3DTi further increased to 107.0 ± 7.8 nm, 123.0 ± 3.0 nm, and 76.3 ± 7.8 nm for 3DTi-FP1, 3DTi-FP2, and 3DTi-NFP, respectively. The increased roughness enhanced the hydrophilicity of the Ti surface, as confirmed by contact angle measurements (Fig. 2F). This enhancement in hydrophilicity facilitates interaction between the implant and surrounding cells, promoting cell adhesion and proliferation [10,45].

The high elastic modulus of Ti implants can adversely affect osseointegration with surrounding bone tissue. Compressive testing of the different samples using a universal testing machine revealed that the elastic modulus of 3DTi was 11.63 ± 3.29 GPa, significantly lower than that of Ti (94.03 ± 6.14 GPa). Notably, the grafting of FPs did not significantly affect the elastic modulus of 3DTi (15.20 ± 2.38 GPa), a value closely aligned with that of human cortical bone (14.4 ± 0.85 GPa) (Fig. 2G).

Overall, through various surface characterization techniques, we demonstrated that a thin, adhesive peptide layer forms rapidly and spontaneously on the 3DTi surface upon simple immersion in the FPs solution. The surface properties of 3DTi remained largely unchanged after peptide conjugation, underscoring the importance of specific Ti-binding peptides in surface adhesion and functionalization. Thus, we successfully developed a simple and efficient strategy for the multifunctional surface engineering of Ti implants.

### 3.4 Biocompatibility and adhesion evaluation of samples

**Fig. 3:**
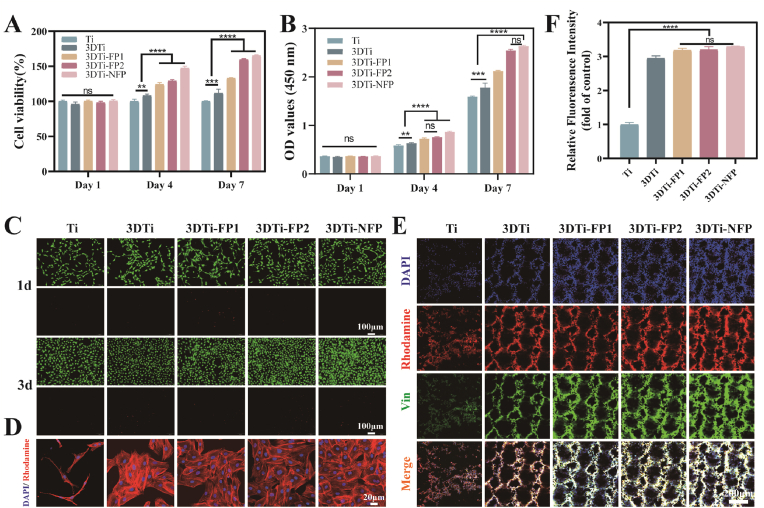
Biocompatibility and adhesion activity of modified Ti substrate. (A–B) CCK-8 assay results show BMSCs proliferation on different sample surfaces. (C) Fluorescence images of live/dead staining of BMSCs cultured on different surfaces. (D) Fluorescence microscope images of cytoskeletal staining of BMSCs. (E) Immunofluorescent staining of vinculin to show focal adhesions of BMSCs on various Ti substrates. (F) Semi-quantitative analysis of immunofluorescent vinculin staining across different samples. Data were presented as mean ± SD. ∗*p* < 0.05, ∗∗*p* < 0.01, ∗∗∗*p* < 0.001, ∗∗∗∗*p* < 0.0001, and ns: no significance.

The interaction between cells and materials is crucial for a material's capacity to enhance bone regeneration. CCK-8 assay, live/dead staining, and immunofluorescence assays were used to evaluate the cytotoxicity, adhesion, and proliferation capacity of BMSCs on different Ti substrates. With extended culture periods, significant increases in cell proliferation were observed, especially on days 4 and 7, highlighting the positive effects of peptides on cell viability and proliferation (Fig. 3A and B). Furthermore, live/dead staining results after 1 and 3 days of culture showed no evident cytotoxicity across the Ti substrates, and cell proliferation was notably enhanced with peptide loading (Fig. 3C), consistent with the CCK-8 findings. These results confirm that the prepared Ti substrates exhibit excellent cytocompatibility and significantly promote cell adhesion and proliferation.

Cell morphology plays a critical role in regulating cellular phenotype. As shown in Fig. 3D, after 4 h of co-culture, BMSCs adhered to all Ti substrates; however, distinct differences in cell morphology were observed. Compared to the control Ti surface, where cells appeared elongated and less numerous, cells on the 3DTi surface displayed a more polygonal shape with enhanced spreading and extensive branched pseudopodia. With peptide loading, cell numbers further increased, showing even more stretched morphologies, well-developed cytoskeletal structures, and branched pseudopodia, with tight intercellular connections confirming the potent regulatory effect of 3DTi and its conjugated peptides on cell adhesion and morphology.

To further validate the affinity of 3DTi and the FPs for cells, we conducted vinculin immunofluorescence staining, as vinculin is commonly used as a marker for focal adhesions [8]. Focal adhesion formation was observed on all 3DTi substrates, indicating good cell affinity due to the hydrophilicity of the conjugated peptides (Fig. 3E). Additionally, the presence of the adhesive peptide GFOGER significantly enhanced the formation of focal adhesions, indicating that these surfaces effectively support cell growth (Fig. 3F). Together, these findings demonstrate that 3DTi loaded with FPs significantly enhances early cell adhesion, proliferation, and spreading, confirming excellent cytocompatibility.

### 3.5 Antibacterial action in vitro

**Fig. 4:**
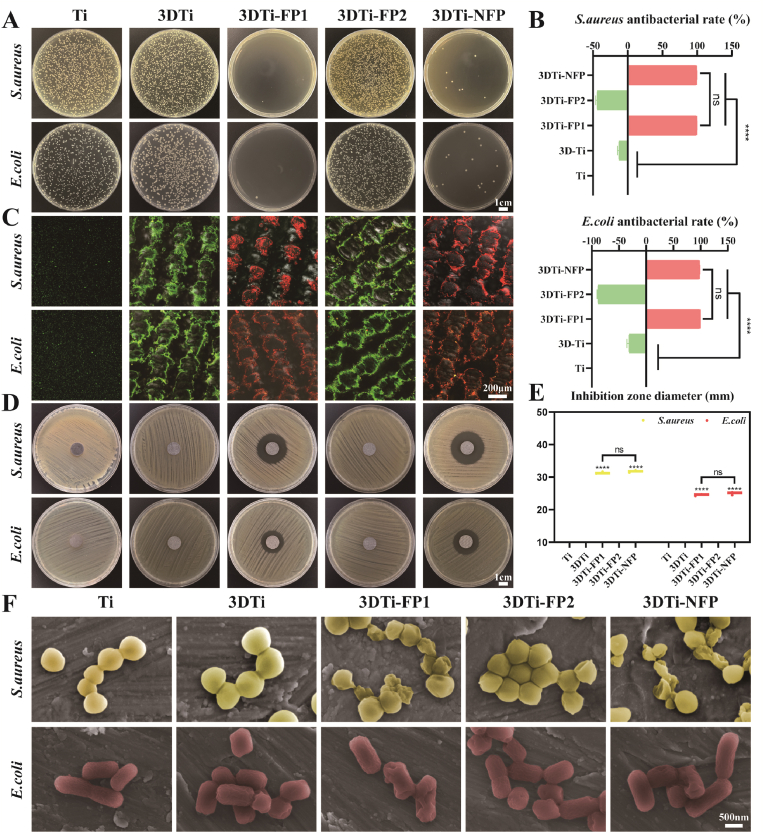
In vitro antibacterial analysis of modified Ti substrates. (A) Representative images of *E. coli* and *S. aureus* colonies on different groups. (B) Quantitative analysis of the antibacterial efficacy (%) against *E. coli* and *S. aureus* across different groups. (C) Representative fluorescence images of live/dead-stained bacteria, with live bacteria in green and dead bacteria in red. (D) Representative images of inhibition zones for *E. coli* and *S. aureus* culture on different groups, with quantitative analysis of inhibition zone diameters shown in (E). (F) Representative SEM images of bacterial morphology on different groups. Data were presented as mean ± SD. ∗*p* < 0.05, ∗∗*p* < 0.01, ∗∗∗*p* < 0.001, ∗∗∗∗*p* < 0.0001, and ns: no significance.

Preventing bacterial colonization and proliferation is a prerequisite for promoting bone formation. Antibacterial effects of the samples against Gram-positive *S. aureus* and Gram-negative *E. coli* were evaluated using colony counting, inhibition zone assays, live/dead staining, and SEM imaging. As shown in Fig. 4A, almost no colony formation was observed in the 3DTi-FP1 and 3DTi-NFP groups, with antibacterial rates exceeding 98 % for *S. aureus* and 90 % for *E. coli* (Fig. 4B). This finding suggests that samples lacking KR-12 are more susceptible to bacterial proliferation. In Fig. 4C, abundant green fluorescence was observed in the Ti, 3DTi, and 3DTi-FP2 groups, indicating good viability of *S. aureus* and *E. coli* on these surfaces, whereas almost all bacteria in the 3DTi-FP1 and 3DTi-NFP groups exhibited red fluorescence, indicating that most bacteria were dead. The inhibition zone assay further corroborated these findings (Fig. 4D). After 12 h of culture, clear inhibition zones formed around the 3DTi-FP1 and 3DTi-NFP groups, while no inhibition zones were observed around the Ti, 3DTi, and 3DTi-FP2 groups, suggesting that KR-12 release possesses potent bactericidal properties. The inhibition zone diameters against *S. aureus* and *E. coli* for the 3DTi-FP1 and 3DTi-NFP groups were 31.35 ± 0.23 mm, 31.76 ± 0.21 mm, 24.52 ± 0.17 mm, and 25.02 ± 0.34 mm, respectively (Fig. 4E). Unexpectedly, no significant differences in inhibition zone size were observed between the 3DTi-FP1 and 3DTi-NFP groups, suggesting that the presence of GFOGER does not affect the antibacterial activity of KR-12.

SEM observations of bacterial morphology and membrane integrity further confirmed these results (Fig. 4F). On the Ti, 3DTi, and 3DTi-FP2 samples, *S. aureus* and *E. coli* exhibited typical spherical and rod shapes with smooth, intact membranes, indicating no adverse effects on bacterial viability. In contrast, bacteria adhered to the 3DTi-FP1 and 3DTi-NFP surfaces displayed irregular shapes, with signs of shrinkage, deformation, and even membrane rupture. These findings clearly demonstrate the potential of KR-12-loaded 3DTi implants to significantly reduce the risk of bacterial infection in vitro. Various engineered KR-12 peptides have been shown to eradicate multidrug-resistant pathogens, such as MDR *Pseudomonas aeruginosa* (MDR-PA) and *methicillin-resistant S*. *aureus* (MRSA) [46], as well as *Acinetobacter baumannii* and *S*. *epidermidis* [47], while effectively disrupting pre-formed biofilms. This supports its potential efficacy against mixed bacterial infections. Given its membrane-disrupting mechanism [18,19] and the sustained release from the 3DTi-NFP surface, it is reasonable to hypothesize that the bactericidal properties of 3DTi-NFP implants remain robust in complex microbial environments, effectively reducing the risk of polymicrobial infections while simultaneously promoting osseointegration.

### 3.6 Osteogenesis in vitro

**Fig. 5:**
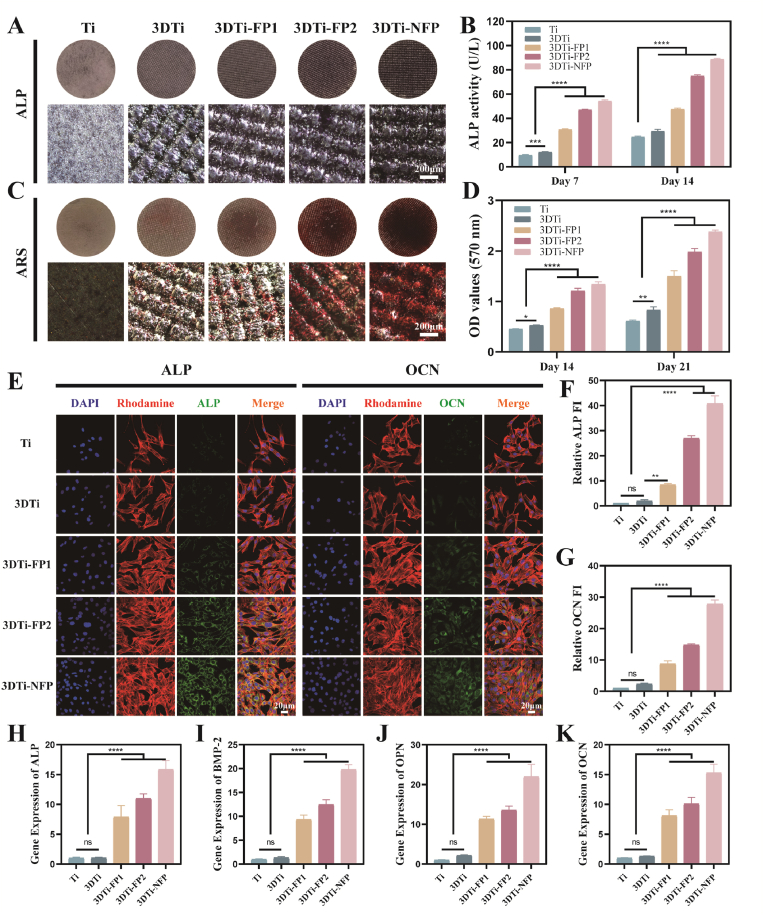
Osteogenesis of different Ti substrates in vitro. (A) Microscopic and optical images of ALP-stained cells after 7 days of culture. (B) Quantitative analysis of cellular ALP activity. (C) Microscopic and optical images of ARS-stained cells after 14 days of osteogenic differentiation. (D) Quantitative analysis of calcium mineralization. (E) Immunofluorescence staining of BMSCs cultured on different Ti substrates, showing the cytoskeleton (red), ALP and OCN proteins (green), and nuclei (blue). Semi-quantitative analysis of immunofluorescence staining for (F) ALP and (G) OCN. (H–K) qRT-PCR analysis of ALP, BMP2, OPN, and OCN gene expression levels in BMSCs cultured on different Ti substrates after 14 days (n = 4). Data were presented as mean ± SD. ∗*p* < 0.05, ∗∗*p* < 0.01, ∗∗∗*p* < 0.001, ∗∗∗∗*p* < 0.0001, and ns: no significance.

Effective osseointegration is a critical strategy to address peri-implant bone defects and implant loosening. To assess the osteogenic potential of different samples on BMSCs, a comprehensive analysis was conducted, including early osteogenic marker ALP activity, late-stage osteogenic marker ARS staining, qRT-PCR, and immunofluorescence staining. Fig. 5A and C display the ALP and ARS staining results after 7 and 14 days, respectively. The 3DTi-NFP group demonstrated the highest staining intensity and density, followed by the 3DTi-FP2 and 3DTi-FP1 groups. By day 14, ALP expression in the 3DTi-NFP group was 3.6 times higher than that of the Ti control group. The ARS staining results showed a similar trend, with the 3DTi-NFP group exhibiting the deepest staining and most abundant formation of mineralized nodules. Semi-quantitative analyses of ALP and ARS staining further confirmed this observation (Fig. 5B and D).

Immunofluorescence staining was used to detect the expression of bone-specific ECM proteins (ALP, BMP-2, OPN, and OCN). After 14 days, the 3DTi surfaces showed high cell density with cells exhibiting a polygonal morphology, characteristic of osteoblasts. As shown in Fig. 5E–G and S5, protein expression in the 3DTi-NFP group was significantly enhanced, with bright green fluorescence, particularly when compared with the other groups, with 3DTi-FP2 ranking next. This suggests a strong role for the GFOGER peptide in promoting osteogenic differentiation, and a synergistic effect of KR-12 with GFOGER that likely enhances osteogenesis by upregulating key osteogenic genes.

To further elucidate the molecular mechanisms underlying osteogenic differentiation, we analyzed the expression levels of key osteogenesis-related genes, including ALP, BMP-2, OPN, and OCN. The results revealed a significant upregulation of these genes on day 14 compared to the control group, with the 3DTi-NFP group showing the highest expression levels (Fig. 5H–K). Additionally, the surface topography of 3D-printed titanium implants plays a crucial role in their interaction with the cellular microenvironment. Previous studies have demonstrated that anodised titania nanotube (TiNT) layers with nanoscale roughness (21–130 nm) can form on 3D-printed titanium surfaces. These TiNT surfaces, with varying pore sizes, promote bone regeneration by modulating immune cell responses by releasing bioactive factors [40]. Based on these findings, we further explored the correlation between surface roughness (Ra) and osteogenic gene expression (Fig. S6). The results indicated a positive correlation between increasing Ra and elevated gene expression levels. This suggests a potential synergistic effect between enhanced surface roughness and increased hydrophilicity due to peptide modification, collectively promoting the expression of osteogenic markers and supporting bone tissue regeneration.

**Fig. 6:**
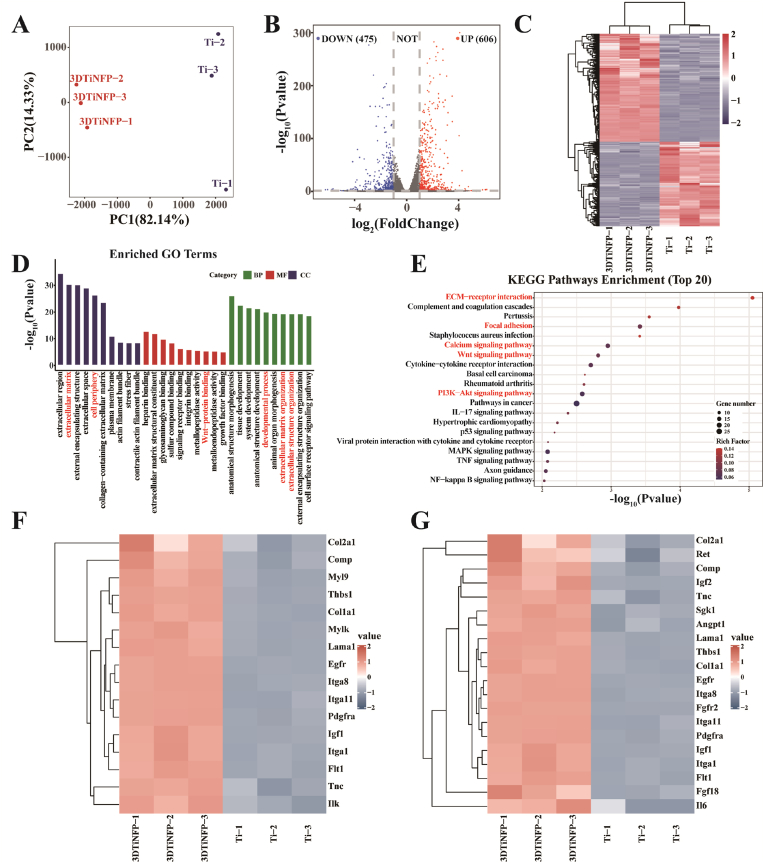
Informatics analysis of gene expression profiles. (A) PCA of different samples in the 3DTi-NFP and Ti control groups. (B) Volcano plot of DEGs comparing 3DTi-NFP vs. Ti. (C) The heat map shows all upregulated and downregulated DEGs in the 3DTi-NFP and Ti groups. (D) Bar chart representing GO enrichment analysis of upregulated DEGs. (E) Bubble plot of upregulated KEGG pathway enrichment terms in 3DTi-NFP vs. Ti; clustered heatmaps display DEGs involved in focal adhesion (F) and PI3K-AKT signaling pathways (G).

To elucidate the mechanism by which 3DTi-NFP promotes osteogenic differentiation of BMSCs, RNA-seq was performed. Principal component analysis (PCA) of the samples revealed a clear separation between the 3DTi-NFP and Ti groups, confirming that both groups met quality control standards (Fig. 6A). Volcano and heat maps of DEGs identified 606 upregulated and 475 downregulated genes in the 3DTi-NFP group compared to the Ti control group (Fig. 6B and C). GO enrichment analysis of the DEGs was categorized into three main domains: biological processes (BP), molecular functions (MF), and cellular components (CC) [48]. Notably, the upregulated genes in the 3DTi-NFP group were significantly enriched in categories such as developmental processes, extracellular matrix organization, integrin binding, Wnt-protein binding, and the extracellular matrix, relative to the Ti group (Fig. 6D).

We performed an enrichment analysis of GOs using the Kyoto Encyclopedia of Genes and Genomes (KEGG) database, identifying the top 20 pathways with the highest enrichment scores (Fig. 6E). These pathways include those related to the positive regulation of cell adhesion, proliferation, and matrix formation, such as focal adhesion and ECM-receptor interaction pathways, as well as pathways closely associated with osteogenesis, including the Wnt signaling pathway, calcium signaling pathway, and PI3K-Akt signaling pathway. Further clustering of these genes was conducted to explore protein-level interactions. As shown in Fig. 6F and G, the genes are involved in cell proliferation and differentiation (e.g., *Igf1*, *Fgfr2*), extracellular matrix components (e.g., *Lama1*, *Col1a1*), and osteogenesis-related processes (e.g., *Col1a1*, *Itga1, Itga11*). These transcriptomic findings are consistent with the in vitro experimental results shown in Fig. 5, indicating that 3DTi-NFP implants may promote new bone formation by enhancing BMSCs adhesion, proliferation, differentiation, and ECM organization through synergistic effects of KR-12 and GFOGER peptides.

### 3.7 In vivo antibacterial and osteogenic synergy enhances osseointegration in infected bone defects

**Fig. 7:**
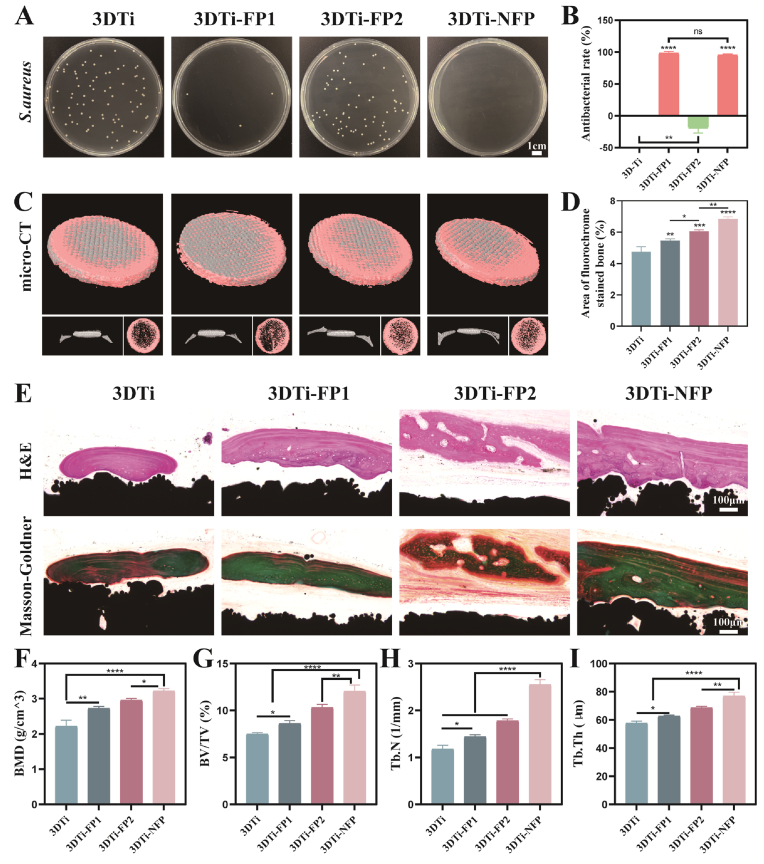
Antibacterial and osteogenic evaluation of 3DTi implants in an *S. aureus*-infected cranial defect model in SD rats. (A) Images of bacterial colonies collected from wound exudates around the 3DTi implant site at 3 days post-surgery; (B) Quantitative analysis of antibacterial efficacy of various 3DTi substrates post-implantation; (C) Micro-CT 3D reconstruction images showing newly formed bone (in pink) surrounding the implants (in gray) (bottom left: coronal CT view; bottom right: top view); (D) Quantitative fluorescence analysis; (E) Histological images of newly formed bone surrounding the implant stained with H&E and Masson-Goldner's trichrome; (F–I) Quantitative micro-CT parameters: BMD, BV/TV, Tb.N, and Tb.Th. (n = 5) Data were presented as mean ± SD. ∗*p* < 0.05, ∗∗*p* < 0.01, ∗∗∗*p* < 0.001, ∗∗∗∗*p* < 0.0001, and ns: no significance.

To evaluate the implants' in vivo antibacterial and osteogenic capabilities, we utilized a cranial defect model with 3DTi-based implants. After creating an 8-mm-diameter critical-size defect in the calvaria of SD rats, implants were placed, and *S. aureus* was injected into the defect site to simulate post-implantation infection. Exudates were collected from the implant sites 3 days post-implantation to assess antibacterial efficacy. Colony count results (Fig. 7A and B) showed a high bacterial attachment in the 3DTi and 3DTi-FP2 groups. In contrast, the 3DTi-FP1 and 3DTi-NFP groups exhibited minimal bacterial growth, with antibacterial rates of 98.49 % and 95.48 %, respectively, consistent with in vitro findings. At 8 weeks post-surgery, micro-CT was used to assess antibacterial and osteogenic performance in rat cranial bone specimens, and the defect area was reconstructed in 3D (Fig. 7C and D). Over time, the 3DTi group displayed limited new bone formation, suggesting that residual *S. aureus* hindered bone regeneration. In contrast, the 3DTi-FP1, 3DTi-FP2, and 3DTi-NFP groups, which were loaded with FPs, showed enhanced bone formation, indicating that KR-12 effectively suppressed *S. aureus* infection. Notably, the bone formation in the 3DTi-NFP group was unaffected by the presence of the adhesion peptide, showing greater bone regeneration than in the 3DTi-FP1 group. This result suggests that the complex in vivo immune environment promotes osteoblast adhesion rather than bacterial attachment. The analysis of key parameters is based on quantitative micro-CT data, including BMD, BV/TV, Tb.N, and Tb.Th, demonstrated the superior performance of 3DTi-NFP. This success may be attributed to the favorable microenvironment created by 3DTi-NFP, which promotes both anti-infection properties and bone synergistic induction for the implants (Fig. 7F–I).

Subsequently, H&E staining and Masson-Goldner trichrome staining were performed to observe the bone formation process over 8 weeks (Fig. 7E). In this staining, green represents trabecular and cortical bone, orange indicates collagen fibers, blue-purple denotes osteocyte nuclei, and black signifies the 3DTi implant. The results revealed a significant presence of collagen fibers between the newly formed bone and the implants in the 3DTi-FP1 and 3DTi-FP2 groups, indicating a mixed repair of fibrous and bone-like tissues. In contrast, the 3DTi-NFP group exhibited more newly mineralized bone, achieving true “osseointegration”.

Importantly, at the end of the 8-week experimental period, the pathological examination of H&E staining revealed no abnormalities in the major metabolic organs (heart, liver, spleen, lung, and kidney) associated with the presence of *S*. *aureus* and peptide-loaded Ti implants. This finding further corroborates the excellent biocompatibility of all implant materials (Figs. S7 and S8).

In summary, these results indicate that the 3DTi-NFP implants effectively inhibit *S*. *aureus* infection while promoting bone healing, demonstrating optimal repair outcomes for cranial defects. The findings include 1) eradication of *S*. *aureus* infection; 2) composition and architecture of newly formed bone resembling that of natural bone tissue, with observable neovascularization; 3) increased coverage of the defect, with a transition from a primarily fibrous structure to bone-like architecture, achieving osseointegration.

The 3DTi-NFP implant, constructed through the peptide platform, effectively enhanced the bioactivity of the implant, addressing the critical challenges of infection and osseointegration commonly encountered by traditional implants. As our understanding of bone regeneration mechanisms deepens, this engineered modification strategy is expected to play a significant role in broader clinical applications. Moving forward, further optimization of peptide sequences to improve targeting and efficacy, alongside the incorporation of the complex biological responses involved in bone regeneration, particularly the regulation of signaling pathways, holds great potential. By integrating gene editing and stem cell technologies, it may be possible to minimize potential side effects and immune reactions, thereby promoting the development of customized implants that better meet specific clinical needs. Crucially, this implant autonomously releases bioactive molecules without the need for external stimuli, making it highly promising not only for applications in orthopedics and dentistry but also in other tissue regeneration fields, such as cartilage repair and nerve regeneration. It holds the potential to become a groundbreaking therapeutic strategy for bone regeneration and tissue repair, significantly contributing to the advancement and innovation in these fields.

## 4 Conclusions

In conclusion, we have successfully incorporated a novel fusion peptide into 3D-printed Ti-based implants, endowing them with specific loading and sustained release capabilities. This approach leverages the synergistic antibacterial and osteogenic properties of the peptide to address the persistent challenges of infection and inadequate osseointegration in biomedical devices. The surface of the 3DTi-NFP implants effectively achieves both sterilization and promotion of bone regeneration, which are crucial for bone healing and enhancing the osseointegration around the implants. Notably, beyond its antibacterial properties, the synergistic effect of the antimicrobial peptide KR-12 and the adhesion peptide GFOGER significantly enhances the osteogenic potential of BMSCs, potentially involving the activation of the focal adhesion and the PI3K-Akt signaling pathway. This mechanism accelerates the regeneration of newly formed bone surrounding the implant. Thus, this study establishes a promising strategy that successfully imparts antibacterial and osteogenic characteristics to 3DTi-based implants, offering significant clinical potential to mitigate surgical complications and implant failures caused by bacterial infections.

## CRediT authorship contribution statement

**Chenying Cui:** Writing – original draft, Visualization, Validation, Methodology, Investigation, Data curation, Conceptualization. **Yifan Zhao:** Methodology, Data curation, Conceptualization. **Jingyu Yan:** Methodology, Investigation, Data curation. **Ziyang Bai:** Data curation. **Guning Wang:** Methodology. **Yingyu Liu:** Methodology. **Yurong Xu:** Methodology. **Lihong Zhou:** Methodology. **Kaifang Zhang:** Methodology. **Yanling Mi:** Methodology. **Binbin Zhang:** Methodology. **Xiuping Wu:** Investigation, Funding acquisition, Conceptualization. **Bing Li:** Investigation, Funding acquisition, Conceptualization.

## Declaration of competing interest

The authors declare that they have no known competing financial interests or personal relationships that could have appeared to influence the work reported in this paper.

## Data Availability

Data will be made available on request.
