# Supplementary material for: Peptide platform for 3D-printed Ti implants with synergistic antibacterial and osteogenic functions to enhance osseointegration
Source: Mater Today Bio. 2024 Dec 26;30:101430. doi: 10.1016/j.mtbio.2024.101430 (PMC11743903; doi:10.1016/j.mtbio.2024.101430)
Supplement: Multimedia component 1 [file mmc1.docx]

Supporting Information

**Peptide platform for 3D-printed Ti implants with synergistic antibacterial and osteogenic functions to enhance osseointegration**

Chenying Cui ^a,b,1^, Yifan Zhao ^a,b,1^, Jingyu Yan ^a,b,1^, Ziyang Bai ^a,b^, Guning Wang ^a,b^, Yingyu Liu ^a,b^, Yurong Xu ^a,b^, Lihong Zhou ^a,b,c^, Kaifang Zhang ^a,b^, Yanling Mi ^a,b^, Binbin Zhang ^a,b^, Xiuping Wu ^a,b,**^, Bing Li ^a,b,*^

^a^ Shanxi Medical University School and Hospital of Stomatology, Taiyuan, 030001, Shanxi, China.

^b^ Shanxi Province Key Laboratory of Oral Diseases Prevention and New Materials, Taiyuan, 030001, Shanxi, China.

^c^ Academy of Medical Sciences, Shanxi Medical University, Taiyuan, 030001, Shanxi, China.

** Corresponding author.

* Corresponding author.

*E-mail addresses:* 77wxp@163.com (X. Wu), [libing1975vip@163.com](mailto:libing1975vip@163.com) (B. Li)

^1^ These authors contributed equally to this work.

**
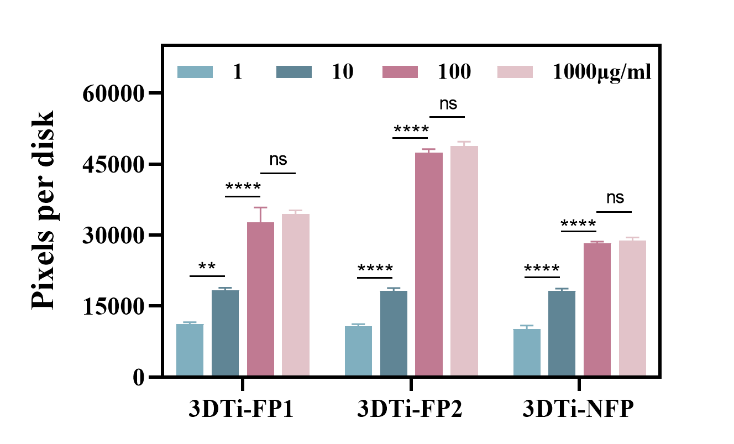
**

**Fig. S1.** Quantification of pixel density for optimal concentration screening of FP1, FP2, and NFP. Data were presented as mean ± SD. **p* < 0.05, ***p* < 0.01, ****p* < 0.001, *****p* < 0.0001, and ns: no significance.

**
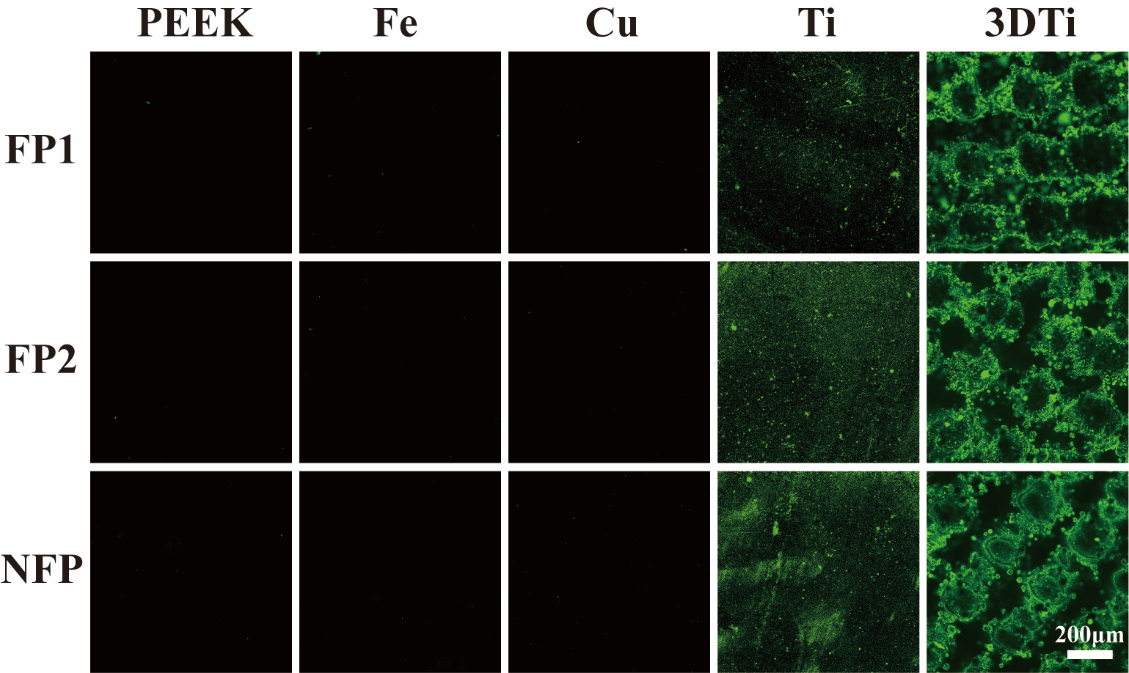
**

**Fig. S2.** The CLSM images of the FITC-FPs combined on both Ti and non-Ti substrates.

**
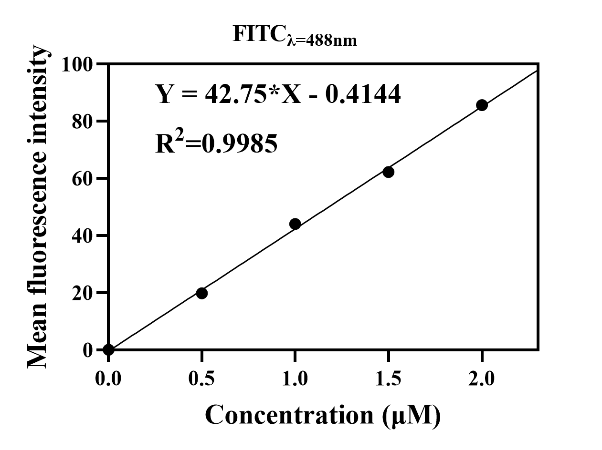
**

**Fig. S3.** FITC- fusion peptides fluorescence standard curve.

**
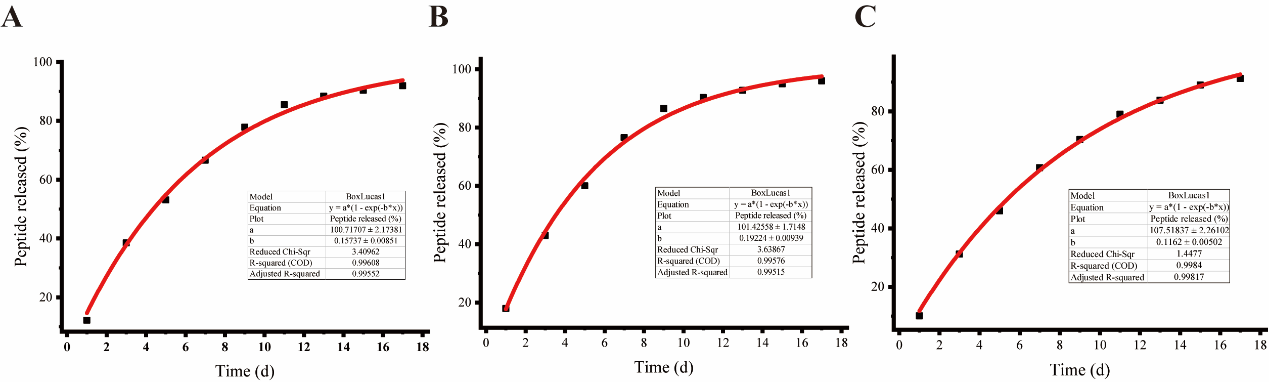
**

**Fig. S4.** (A) FITC-FP1 peptide release kinetics fitting curve. (B) FITC-FP2 peptide release kinetics fitting curve. (C) FITC-NFP peptide release kinetics fitting curve.


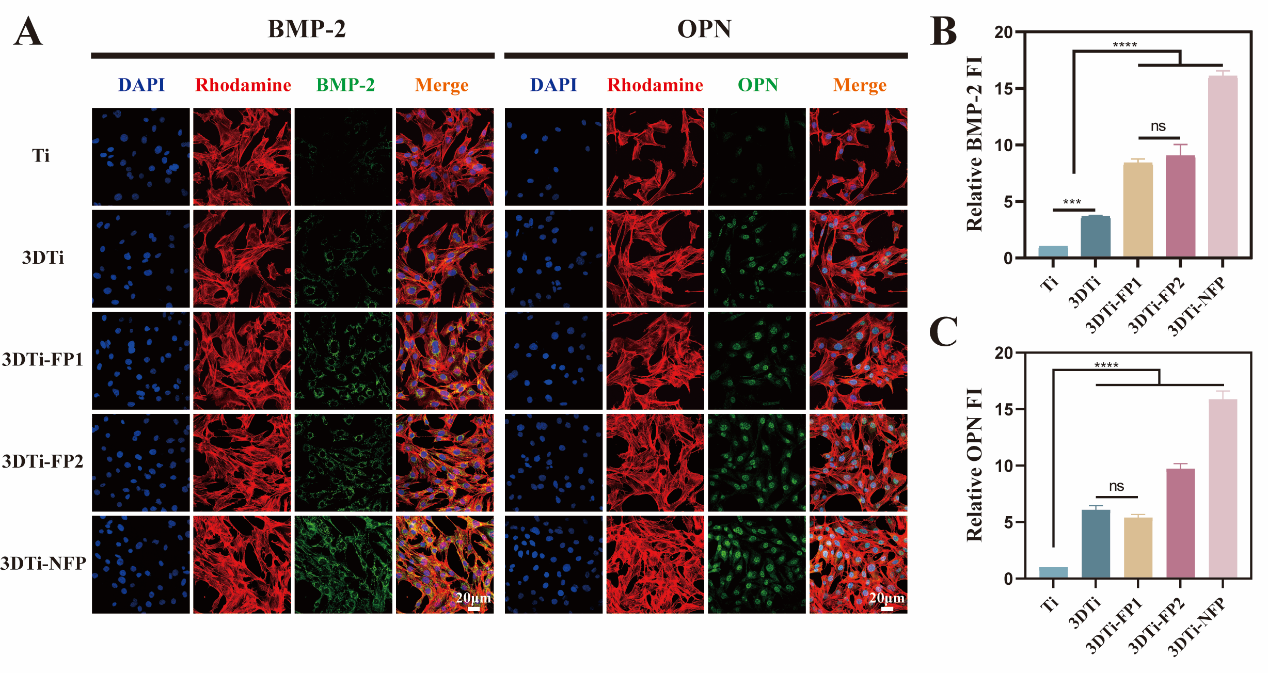


**Fig. S5.** (A) Immunofluorescence staining of BMSCs cultured on different Ti substrates, showing the cytoskeleton (red), BMP-2 and OPN proteins (green), and nuclei (blue). Semi-quantitative analysis of immunofluorescence staining for (B) BMP-2 and (C) OPN. Data were presented as mean ± SD. **p* < 0.05, ***p* < 0.01, ****p* < 0.001, *****p* < 0.0001, and ns: no significance.

**
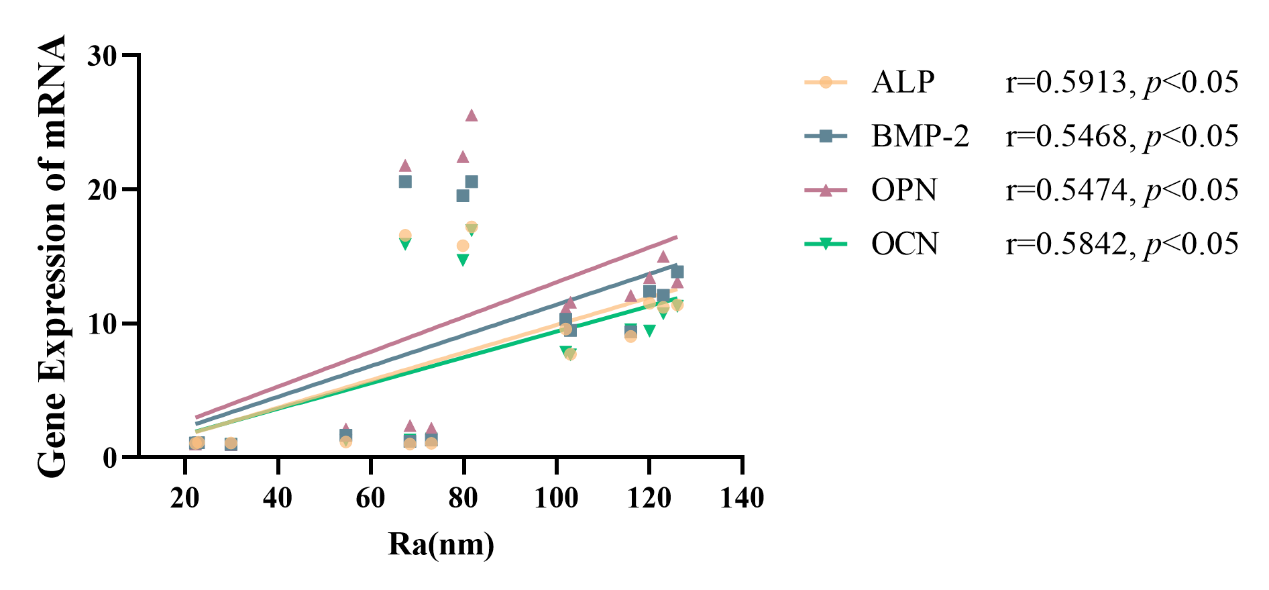
**

**Fig. S6.** Correlation analysis and fitting between surface roughness of different modified Ti substrates and expression of osteogenic genes.


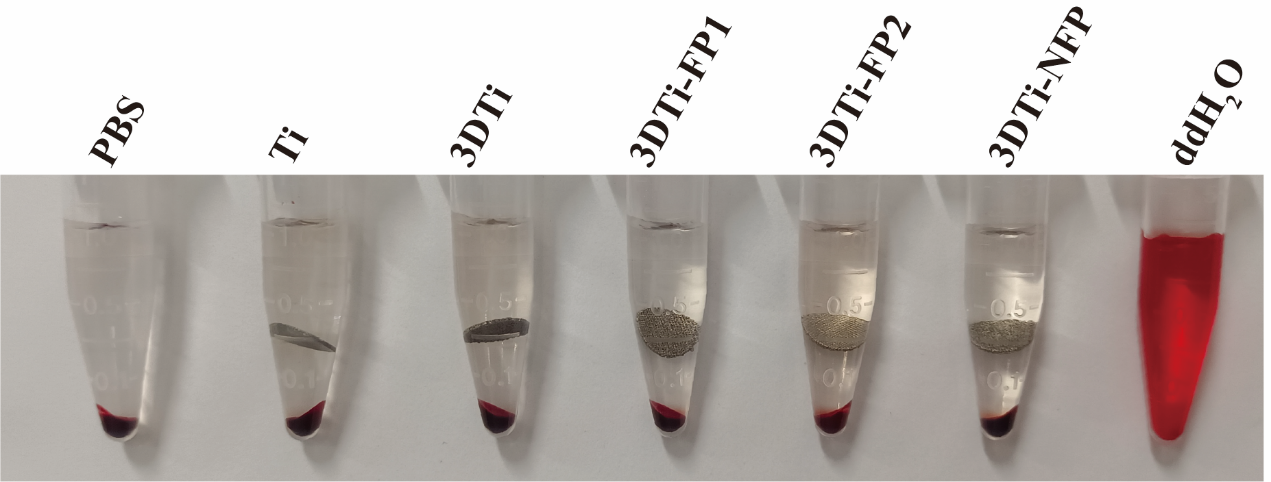


**Fig. S7.** Performance of different fusion peptide-loaded 3D Ti implants in hemolysis assays.


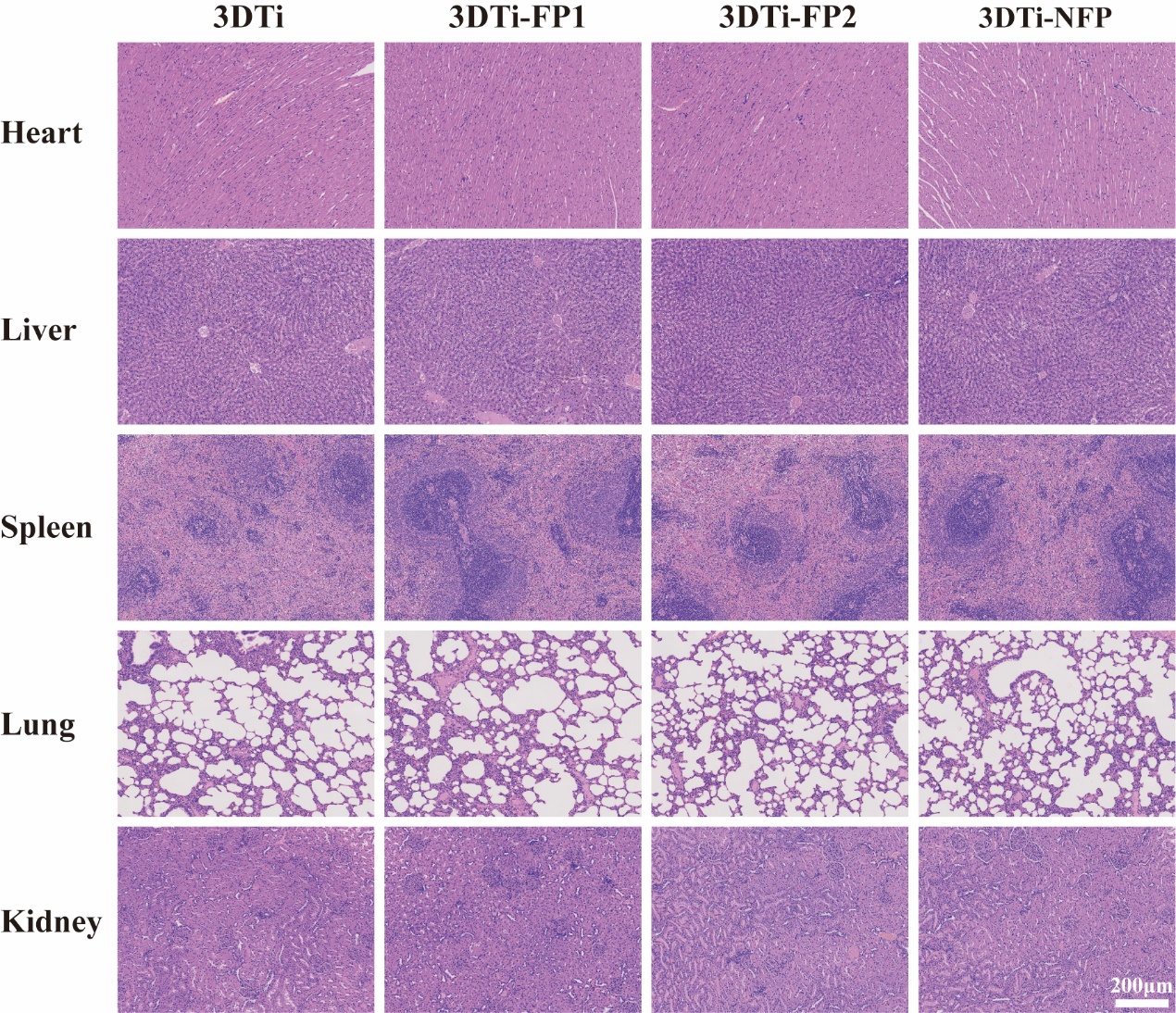


**Fig. S8.** H&E staining of heart, liver, spleen, lung, and kidney.

**Table S1. The sequence information for the three fusion peptides.**

| Name | Composition | Sequences | Molecular Weight |
| --- | --- | --- | --- |
| FP1 | minTBP-1+Linker+ KR-12 | RKLPDAAEAAAKEAAAKAKRIVQRIKDFLR | 3335.95Da |
| FP2 | minTBP-1+Linker+GFOGER | RKLPDAAEAAAKEAAAKAGFOGER | 2441.73Da |
| NFP | minTBP-1+Linker+Lys+GFOGER+KR-12 | RKLPDAAEAAAKEAAAKA[K(GFOGER)]KRIVQRIKDFLR | 4123.84Da |

**Table S2. Surface element content percentages of different samples determined by XPS.**

| Samples | Element content percentages (%) | | | |
| --- | --- | --- | --- | --- |
|  | C | N | O | Ti |
| Ti | 85.15±1.89 | 0.33±0.36 | 13±0.74 | 1.52±1.33 |
| 3DTi | 49.35±0.73 | 2.35±1.37 | 39.59±1.38 | 8.71±0.31 |
| 3DTi-FP1 | 77.29±2.36 | 6.83±1.48 | 15.14±2.65 | 0.74±1.89 |
| 3DTi-FP2 | 86.72±1.38 | 3.54±0.64 | 9.03±0.26 | 0.71±0.29 |
| 3DTi-NFP | 83.89±2.45 | 7.04±1.65 | 8.61±0.17 | 0.46±0.95 |

**Table S3. The primer sequences used in qRT-PCR.**

| Gene | Forward primer sequence (5'-3') | Reverse primer sequence (3'-5') |
| --- | --- | --- |
| ALP | GACCTCCTCGGAAGACACTC | TGAAGGGCTTCTTGTCTGTG |
| BMP-2 | AACACTGTGCGCAGCTTCC | CTCCGGGTTGTTTTCCCAC |
| OPN | ATGATGGCCGAGGTGATAGT | ACCATTCAACTCCTCGCTTT |
| OCN | GTGCAGAGTCCAGCAAAGGT | TCCCAGCCATTGATACAGGT |
| GAPDH | GACTCATGACCACAGTCCATGC | AGAGGCAGGGATGATGTTCTG |
